# Unsupervised machine learning for identifying morphological phenotypes in abdominal aortic aneurysms using fully automated volume-segmented imaging: a multicentre cohort study

**DOI:** 10.1093/ehjdh/ztaf136

**Published:** 2025-11-18

**Authors:** Michal Kawka, Caroline Caradu, Ruth Scicluna, Colin Bicknell, Matthew J Bown, Manj Gohel, Janet T Powell, Anna L Pouncey

**Affiliations:** School of Health and Medical Sciences, City St George’s University of London, London, UK; St George’s Vascular Institute, St George’s Hospital, London, UK; Vascular Surgery Department, Bordeaux University Hospital, Bordeaux, France; Department of Cardiovascular Sciences and NIHR Leicester Biomedical Research Centre, University of Leicester, Leicester, UK; Department of Surgery and Cancer, Imperial College London, Ayrton Rd, South Kensington, London SW7 5NH UK; Department of Cardiovascular Sciences and NIHR Leicester Biomedical Research Centre, University of Leicester, Leicester, UK; Department of Vascular Surgery, Cambridge University Hospitals, Cambridge, UK; Department of Surgery and Cancer, Imperial College London, Ayrton Rd, South Kensington, London SW7 5NH UK; School of Health and Medical Sciences, City St George’s University of London, London, UK; St George’s Vascular Institute, St George’s Hospital, London, UK; Department of Surgery and Cancer, Imperial College London, Ayrton Rd, South Kensington, London SW7 5NH UK

**Keywords:** Artificial intelligence, Machine leaning, Abdominal aneurysm, Automated imaging analysis

## Abstract

**Aims:**

Thrombo- and microembolic complications following abdominal aortic aneurysm (AAA) repair are hypothesized to be associated with wall thrombus burden. Fully automatic volume segmentation (FAVS) of imaging enables extraction of morphological features from which thrombogenic phenotypes may be identified.

**Methods and results:**

This was a multi-centre retrospective cohort study using FAVS to examine pre-operative imaging for elective AAA repairs (2013–23). Radiological data were matched with National Vascular Registry thromboembolic outcomes data (cerebral, bowel, renal or limb ischaemia). Principal component analysis was used for dimensionality reduction, followed by unsupervised machine learning with *k*-nearest neighbours clustering, with number of clusters determined using silhouette scores. Clusters were compared using multivariate logistic regression, adjusting for aortic size index, cardiovascular risk parameters, and repair-type. Of 1655 patients, 1455 had sufficient quality imaging for FAVS (145 women and 1310 men). *k*-nearest neighbours clustering identified two morphological subtypes (*n* = 878 and *n* = 577), with sex imbalance (13.8 vs. 4.1% women, *P* < 0.001). The clusters differed in wall thrombus burden in visceral vessels, infra-renal aorta, aneurysmal neck, and common iliac arteries (*P* < 0.001). On adjusted multivariate regression, there was no significant differences in thromboembolic events between clusters, although event rate was low (*n* = 31, 2.1%) (odds ratio 1.56, 95% confidence interval 0.71–3.43, *P* = 0.23).

**Conclusion:**

Unsupervised machine learning can identify distinct aneurysm morphological phenotypes with significant thrombus burden difference, which exhibit sex imbalance. While thromboembolic events were infrequent and did not differ significantly between clusters, these anatomical phenotypes may provide a framework for future studies investigating embolic risk and sex-specific disease mechanisms.

## Graphical Abstract

**Graphical Abstract:**
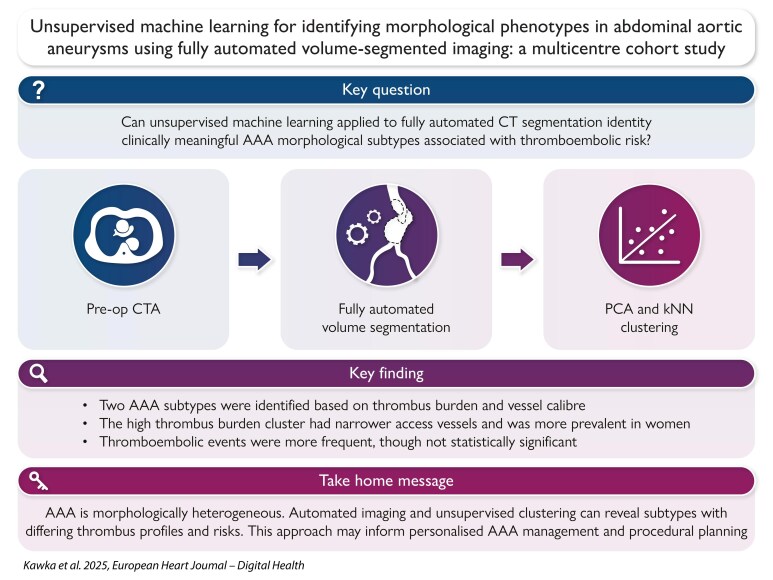

## Introduction

Abdominal aortic aneurysms (AAA) encompass a morphologically heterogenous group of aneurysmal diseases, characterized by various levels of vascular remodelling both within the aneurysm and adjacent visceral, and access vasculature.^1^ Current decision-making around AAA repair is multifactorial, incorporating patient fitness, comorbidities, and patient preferences, alongside anatomical considerations derived from pre-operative imaging. For endovascular aortic repair (EVAR), adherence to device-specific instructions for use dependent on aneurysm morphology remains a key for eligibility.^2^ These traditional anatomical metrics such as vessel diametres still dominate risk stratification and procedural planning. However, these conventional measurements may not fully capture the complexity of aneurysm morphology or accurately predict the risk of adverse outcomes.^3^ Thrombo- and microembolic events causing cerebral, renal, bowel, or limb ischaemia are often catastrophic sequalae of AAA repair, but are difficult to predict.^4^ It has been suggested that higher intraluminal thrombus burden, together with narrower more tortuous arteries of organ supply, increase vulnerability to thromboembolic and microembolic complications.^5^ However, there remains a paucity of evidence regarding risk factors for these events, and potential strategies for their prevention. Furthermore, accurate quantification of thrombus burden and other three-dimensional morphological parameters using manual techniques is time-consuming and labour-intensive, limiting their routine use in clinical decision-making.^6,7^

Our reliance on simple measurements are partly due to limitations of current technique used in pre-operative planning, which is most commonly based on computed tomography angiography (CTA) scanning, enabling the assessment of vascular morphology. Optimal planning for AAA repair requires thin-slice, single arterial-phase CTA (≤1 mm), allowing orthogonal centreline-based diameter measurements.^8^ These conventional measurements remain the clinical standard for both endovascular and open repair. However, volumetric assessment, though not yet part of routine pre-operative planning, is increasingly applied in post-EVAR surveillance, where sac volume changes can inform follow-up strategies. Fully automatic volume segmentation (FAVS) has been shown to be accurate, reproducible, and fast for assessment of AAA diameters and volume.^7^ What is more, FAVS results in rich radiomic datasets, which yield itself to analysis using machine-learning methods, to elucidate complex interaction between geometric and volumetric variables. Comprehensive assessment of FAVS could enable a detailed assessment of vascular anatomy, including additional features such as thrombus burden. These, in line, could be used to identify morphological subtypes amongst aneurysms, which when compared, could help in the identification of additional morphological features associated with adverse outcomes. It is possible therefore that the use of these more advanced morphological assessments may better predict the risk of adverse AAA-related events, such as thrombo- and micro-embolism, than is currently achieved using conventional metrics.

The primary aim of this study was to use FAVS of computed tomography (CT) imaging to investigate the existence of morphological subtypes amongst AAA, comparing them in terms of anatomical differences and differences in patient factors. The secondary aim was to compare the resulting morphological subtypes with regards to clinical outcomes.

## Methods

### Study design and setting

This was an observational retrospective cohort study conducted in three tertiary vascular centres based in the UK. All adult patients over 50 years old, receiving primary open or EVAR for infra-renal AAA or juxta-renal AAA at a participating centre from 1 January 2013 to 7 January 2023 were included. Pre-operative CT imaging was extracted from participating centres. Patient demographic information, co-morbid status, indication for surgery, and outcomes were obtained locally from contemporaneously collected UK National Vascular Registry (NVR) records. Pre-existing variables are defined within the UK NVR data dictionary^9^ and the OPCS Classification of Interventions and Procedures.^10^

Exclusion criteria were: (i) insufficient CT imaging quality to enable automated segmentation (>1 mm slices) or lack of CT imaging within 1 year of operation; (ii) ruptured aneurysm, aorto-iliac occlusive disease, penetrating aortic ulcer, dissection, or supra-renal AAA; (iii) secondary AAA repair; (iv) patients with insufficient clinical data to enable risk stratification (e.g. lack of data regarding co-morbid status). Aortic size index (ASI) was defined as AAA diameter/body surface area (cm/m^2^). Assessment of validity and correction and/or imputation of missing clinical data fields were performed locally by a member of the direct care team during clinical data extraction and pseudonymization.

Fully automatic segmentation software was provided by PRAEVAorta, Nurea® which has received Ce marking (Class IIb).^11^ The use of PRAEVAorta for FAVS has been shown to be fast and reproducible for evaluation of the infra-renal aorta both pre- and post-operatively.^6,7,12^ It has been validated in external cohort and has shown excellent concordance with manual measurements (Dice similarity coefficient >0.9), and fast segmentation time (ranging from 27 s to 4 min per patient).^6,7,12^ Quality assurance for automatic imaging segmentation was conducted in three stages. First, each scan was reviewed by a vascular surgeons not involved in the software development to confirm aneurysm presence and pre-operative status. Second, the resulting three-dimensional reconstructions were visually inspected to verify correct identification of the aorta and visceral branches. Finally, missing measurement fields and outlier values were assessed, and cases with segmentation failure (>10% missing data fields) or inaccurate vessel identification were excluded. Review of the initial clustering outputs was performed independently by vascular surgeons with expertize in complex aortic repair, blinded to all clinical outcomes.

### Outcome measures

Primary outcome measures were vascular morphology: neck morphology (e.g. diameter, length, supra-renal angle, and infra-renal angle), visceral and access vessel diameters, tortuosity index, calcification volume, wall thrombus index, thrombus volume, and maximum common iliac artery stenosis. Wall thrombus index was defined as a proportion of thrombus volume compared with vessel volume at the level measured.

Secondary outcome measures were major post-operative ischaemic complications, defined as a composite endpoint including bowel ischaemia, limb ischaemia, and cerebral ischaemia events recorded during the index admission. These data were obtained from contemporaneous entries in the UK NVR and cross-checked by the local vascular clinical teams.

### Data analysis

All analyses, including machine-learning modelling were conducted using R statistical software (version 4.2.3).^13^ A list of packages and example code utilized can be found in Supplementary material online, *Table S1*. Missing data patterns were explored with assessment of the associations between missing variables, patient sex, and repair modality, and Upset plots illustrating intersections of missingness were created.^14,15^ Clinical variables with insufficient data quality (defined as >25% missing data), were excluded from further analyses. Due to observed difference in the degree of missingness between the sexes, imputation missing data were performed using a *K*-nearest neighbours (KNN) approach, with the comparison of original and imputed data.^16,17^ To maintain methodological consistency and preserve inter-variable correlations, a single KNN imputation strategy was applied across all retained variables.

Following imputation, highly correlated variables (>0.9) were removed, and principal component analysis (PCA) was performed to achieve further dimensionality reduction.^18^ Contribution of each principal component was assessed quantitatively. Initial number of principal components was defined as number of variables divided by 2 (*n*/2), following which it was optimized through sequential trials, to achieve the highest silhouette score. Following PCA, machine-learning approaches were used for clustering. Clinical covariates such as age, sex, and co-morbidity status were intentionally excluded from the clustering input to allow the identification of purely anatomical phenotypes based solely on imaging-derived variables, thereby avoiding bias from predefined clinical features. Supervised machine-learning (ML) approaches were discounted, due to low thromboembolic event rate, and risk of overfitting—instead unsupervised methods were favoured. These unsupervised ML methods included KNN, density-based spatial clustering of applications with noise (DBSCAN), and Gaussian mixture model.^19–21^ Cluster quality was evaluated using silhouette score, with approach yielding highest silhouette score whilst utilizing fewest possible clusters used for further analysis. Cluster separation was also inspected visually using Uniform Manifold Approximation and Projection (UMAP) plots in two-dimensional scape, and through repeated analysis using agglomerative hierarchical clustering with Ward linkage, assessing concordance with the *k*-means solution via adjusted Rand index. Following unsupervised clustering, the anatomical profiles of each cluster, and variables with highest weights on PCA were independently reviewed by two vascular surgeons involved in the study. This review focused on the clinical recognizability of the patterns, particularly in relation to access vessel anatomy, aneurysm extent, and planning considerations for endovascular repair. This was done on a cluster basis and not scan-by-scan basis.

Following clustering, standard parametric and non-parametric testing was then used to assess for inter-cluster differences in the pre-specified variables of interest, with a *P*-value of <0.05 considered significant. No adjustments were made for multiple significance testing (e.g. Bonferroni or false discovery rate); however, grouping of ‘familiar’ variables (e.g. visceral vessel diameters and calcification volumes) was used to guide the level of confidence, with greater weight given to consistent findings across a ‘familiar’ variable group.^22^ This was to allow for detection of signals in this exploratory analysis and follows previously outlined methodology.^22^  *Post hoc* linear regression model was used to determine the effect of demographic variables with significant inter-cluster differences on cluster membership, using McFadden’s *R*^2^.

For secondary outcomes analysis first, cluster-stratified univariate analyses to examine the relation between each variable and the outcome of interest (thromboembolic event) were conducted. A threshold of *P* < 0.1 or a pre-determined high likelihood of clinical or mechanistic significance was used to govern subsequent selection for multivariate analyses. Assessment of multicollinearity, using variance inflation factors were used to determine final variable selection, with a value >2 deemed significant.^23^

The effect of cluster as the exposure was examined on the log odds of a major thromboembolic event and was defined as the average treatment effect on the treated, namely the causal effect of cluster on the outcome, and the within approach was utilized, meaning that matching and estimates of casual effects and standard errors were performed within each imputed dataset followed by pooling of the results.

### Regulatory aspects and ethical approval

The project was approved by the Health Research Authority (HRA) and Health and Care Research Wales (IRAS: 292985, REC: 21/HRA/0498), and sponsored by Imperial College London. Data processing agreements and research service agreements were agreed by Nurea (company number RCS Bordeaux 841437411 00017, France) and Imperial College of Science, Technology and Medicine, which acted as a sponsor for the study. Digital Imaging and Communications in Medicine (DICOM) data were pseudonymized by local care teams through the removal of patient identifiable/personal meta-data and replacement with a study identification number. All data for the project were stored securely on a password protected, General Data Protection Regulation (GDPR) compliant, secure cloud server, owned by Imperial College London, or on encrypted hard drives, stored in a locked location at Imperial College London. Pseudonymized DICOM data were securely transferred to Nurea’s server for FAVS, following which, an output report was created and DICOM data were deleted. Nurea was not party to any identifiable, sensitive, or personal data. This study was conducted according to the Strengthening the Reporting of Observational Studies in Epidemiology (STROBE) and REporting of studies Conducted using Observational Routinely-collected health Data statement (RECORD) guidelines for observational studies^24^  ^,25^ (see Supplementary material online, *Table S2*).

## Results

### Demographics and cohort characteristics

**Figure 1:**
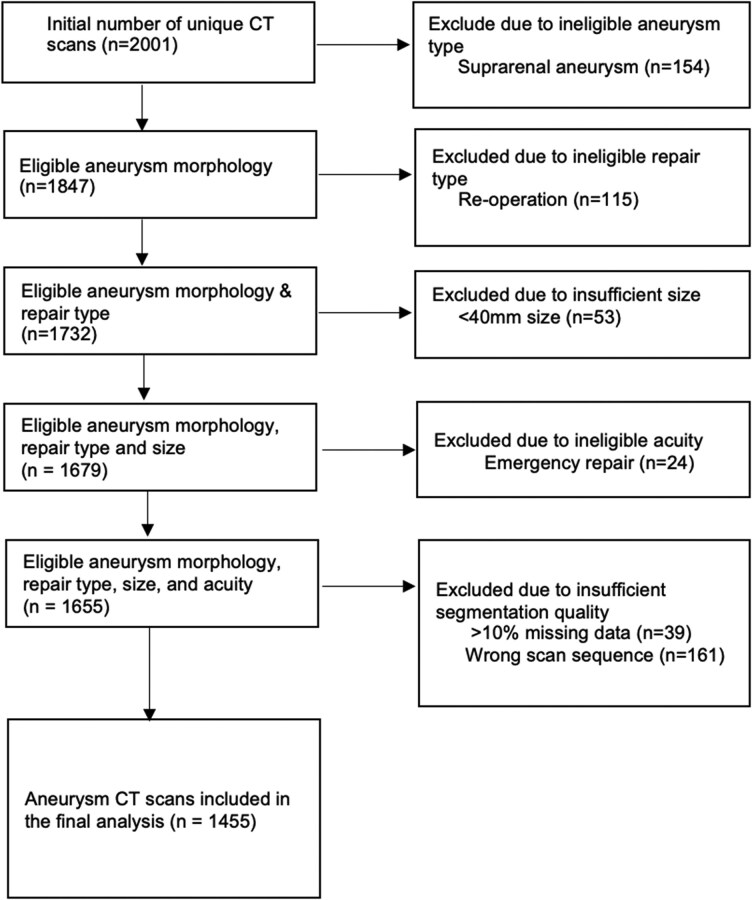
Flow diagram demonstrating patient selection. AAA, abdominal aortic aneurysm; EVAR, endovascular aortic repair.

A total of 1655 patients at the 3 centres met all inclusion criteria, and 1455 had CTA imaging of sufficient quality for analysis. Of the 200 patients excluded, 161 had scan sequence insufficient for segmentation (e.g. non-contrast or >1 mm slice thickness protocol), whilst 39 scans failed segmentation due to software limitation. Of these, a total of 452 patients (40 women, 412 men) received an open repair and 1003 patients (105 women, 892 men) received an EVAR (*Figure 1*).

### Clustering of morphological subtypes and cluster characteristics

**Figure 2:**
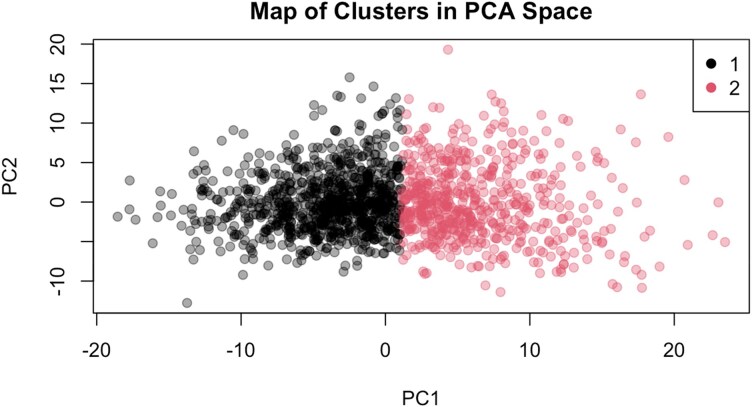
UMAP plot for clusters. Cluster 1 in black, Cluster 2 in red. PC, principal component.

Ten variables were removed from the analysis, due to high percentage of data missingness; all of them were functionally represented elsewhere in the dataset, as the software generates largely duplicate ways of measuring related concepts (e.g. mean and median stenosis). Following PCA, two principal components yielded most accurate cluster separation across all methods of clustering, out of which KNN clustering with two clusters has achieved highest silhouette score, and most pronounced separation of visual inspection of the UMAP plots in two-dimensional space (0.4, Supplementary material online, *Figure S1*). The algorithm generated two clusters; Cluster 1 (*n* = 878) and Cluster 2 (*n* = 557). Despite a moderate average silhouette score (0.4), cluster separation was visually evident in PCA space (*Figure 2*), and robustness was supported by alternative clustering approaches, with cluster membership remaining moderately stable across methods (adjusted Rand index = 0.498).

Cluster 1 had significantly more women (13.8 vs. 4.2%, *P* < 0.001), lower median age (74 vs. 76, *P* = 0.002), and higher proportion of patients with chronic obstructive pulmonary disease (COPD; 28.2 vs. 20.1%, *P* = 0.001). There were no significant differences between clusters in the proportion of patients receiving antiplatelet therapy, anticoagulation, or statins at baseline. Full baseline characteristics of the clusters are presented in *Table 1.* In *post hoc* logistic regression, both sex and age were independently associated with morphological phenotype assignment. Female sex was strongly associated with the Cluster 1 membership [OR 3.83, 95% confidence interval (CI) 2.48–6.15, *P* < 0.001], while increasing age was associated with a lower likelihood of such membership (OR 0.98 per year, 95% CI 0.96–0.99, *P* = 0.0017). Despite these significant associations, the model explained only ∼2.6% of the variance in cluster assignment (McFadden’s = 0.026), indicating that age and sex difference alone do not account for the observed clustering. Full logistic regression model can be found in Supplementary material online, *Table S3*.

**Table 1:** Baseline characteristics between clusters

	Cluster 1	Cluster 2	*P*-value
*n*	878	577	
Age [median (IQR)]	74.00 [69.00, 79.00]	76.00 [69.70, 80.60]	0.002
Female, *n* (%)	121 (13.8)	24 (4.2)	<0.001
Diabetes, *n* (%)	120 (13.7)	89 (15.4)	0.391
HTN, *n* (%)	635 (72.3)	390 (67.6)	0.061
COPD, *n* (%)	248 (28.2)	116 (20.1)	0.001
IHD, *n* (%)	293 (33.4)	175 (30.3)	0.247
HF, *n* (%)	44 (5.0)	37 (6.4)	0.306
CKD, *n* (%)	87 (9.9)	58 (10.1)	1
Stroke, *n* (%)	57 (6.5)	26 (4.5)	0.138
Cancer, *n* (%)	37 (4.2)	28 (4.9)	0.655
PAD, *n* (%)	16 (1.8)	5 (0.9)	0.204
ASA (%)			0.515
1	5 (0.6)	2 (0.3)	
2	160 (18.2)	118 (20.5)	
3	668 (76.2)	421 (73.1)	
4	43 (4.9)	35 (6.1)	
5	1 (0.1)	0 (0.0)	
Hypalbuminaemia, n(%)	122 (22.3)	102 (29.1)	0.028
Anaemia, *n* (%)	192 (25.8)	153 (30.8)	0.064
Abnormal ECG, *n* (%)	210 (24.2)	167 (29.3)	0.039
Smoking status, *n* (%)			0.004
Current smoker	207 (23.7)	99 (17.2)	
Non-smoker	550 (62.9)	375 (65.2)	
Ex-smoker	118 (13.5)	101 (17.6)	
Statin, *n* (%)	707 (80.5)	454 (78.7)	0.430
Beta-blocker, *n* (%)	259 (29.5)	172 (29.8)	0.946
ACEi, *n* (%)	276 (31.4)	175 (30.3)	0.698
Anticoagulation, *n* (%)			0.244
0 agents	148 (16.9)	108 (18.7)	
1 agent	723 (82.3)	460 (79.7)	
2+ agents	7 (0.8)	9 (1.6)	
Antiplatelet, *n* (%)	716 (81.5)	445 (77.1)	0.057
ASI [median (IQR)]	3.14 [2.86, 3.57]	3.13 [2.76, 3.59]	0.274
AAA size [median (IQR)]	61.71 [58.59, 66.95]	63.67 [59.47, 71.90]	<0.001
Symptomatic AAA, *n* (%)	70 (8.0)	32 (5.5)	0.095
Repair-type, *n* (%)			0.370
Open	275 (31.3)	177 (30.7)	
EVAR	517 (58.9)	330 (57.2)	
Complex EVAR	86 (9.8)	70 (12.1)	
MACE, *n* (%)	42 (4.8)	18 (3.1)	0.154
MACED, *n* (%)	46 (5.2)	22 (3.8)	0.257
Mortality, *n* (%)	14 (1.6)	8 (1.4)	0.921
Thromboembolic events, *n* (%)	23 (2.6)	10 (1.7)	0.352
MAE, *n* (%)	68 (7.7)	39 (6.8)	0.547
Centre, *n* (%)			0.706
Cambridge	482 (54.9)	308 (53.4)	
Imperial	107 (12.2)	67 (11.6)	
Leicester	289 (32.9)	202 (35.0)	

ACE-i, angiotensin-converting enzyme-inhibitors; HTN, hypertension; COPD, chronic obstructive pulmonary disease; IHD, ischaemic heart disease; HF, heart failure; CKD, chronic kidney disease; PAD, peripheral artery disease; ASA, American Society of Anaethesiologists; ASI, aortic size index; EVAR, endovascular aortic repair; MACE, major adverse cardiac events; MACED, major adverse cardiac events and death; MAE, major adverse events.

Cluster analysis is shown in *Figure 2*. Upon analysis of PCA and relative weights of variables in each principal component (PC), the top 10 variable contributors in PC1 were related to access vessel diameter (right and left external iliac artery and CIA lumen diameter), whilst in PC2 these were related to aortic neck or juxta-renal diameter. Top PCA variable contribution data are available in *Table 2*.

**Table 2:** Top contributors to Principal Component 1 and Principal Component 2

	PC1	PC2
**Top contributors to Principal Component 1**		
Left CIA mean diameter	0.75	−0.22
Right EIA mean diameter	0.74	−0.16
Left CIA minimum diameter	0.72	−0.24
Left CIA lumen mean diameter	0.72	−0.21
Left CIA cross sectional mean diameter	0.71	−0.16
Right CIA lumen mean diameter	0.71	−0.22
Left CIA max diameter	0.70	−0.14
Left CIA lumen max diameter	0.70	−0.11
Left CIA lumen minimum diameter	0.70	−0.22
Right CIA mean diameter	0.70	−0.24
**Top contributors to Principal Component 2**		
Neck maximum diameter	0.31	0.78
Juxta-renal aorta max diameter	0.38	0.76
Infra-renal aorta lumen diameter	0.35	0.73
Neck mean diameter	0.29	0.72
Neck minimum diameter	0.29	0.72
Juxta-renal aorta lumen mean diameter	0.43	0.71
Visceral aorta mean diameter	0.40	0.70
Neck lumen mean diameter	0.37	0.69
Infra-renal aorta mean diameter	0.29	0.67
Neck lumen mean diameter	0.38	0.65

CIA, common iliac artery; EIA, external iliac artery; PC, principal component.

### Assessment of inter-cluster difference in vascular morphology

Clusters differed significantly in vascular morphology (*Table 3*). Patients in Cluster 1 had narrower (*P* < 0.001), and shorter aneurysmal necks (*P* = 0.005), narrower visceral and access vessels (*P* < 0.001), more stenosed CIAs (*P* < 0.001). There were no clear patterns in vessel tortuosity index. Patients in Cluster 2 had significantly more calcification in both their internal iliac arteries (IIA)s, right CIA, and aneurysmal neck (*P* < 0.05).When wall thrombus index was compared, Cluster 1 had significantly higher wall thrombus index in visceral, infra-renal, and aneurysmal neck, as well as both CIA vessels (*P* < 0.01) (*Table 4*). This is despite there being significantly higher thrombus volume in all those vessels in Cluster 2 (*P* < 0.001). Moreover, there was significantly more thrombus in right IIA amongst Cluster 2 patients (*P* < 0.01).

**Table 3:** Comparison of vascular morphology for Cluster 1 and Cluster 2

	Cluster 1	Cluster 2	*P*-value
*n*	878	577	
**AAA neck [median (IQR)]**			
Neck diameter (mm)	23.64 (21.44, 26.31)	25.64 (23.44, 28.69)	<0.001
Maximum neck diameter (mm)	28.72 (26.05, 31.91)	31.11 (28.40, 34.93)	<0.001
Minimum neck diameter (mm)	21.94 (20.05, 24.25)	23.66 (21.81, 26.47)	<0.001
Neck length (mm)	27.20 (17.39, 37.89)	29.76 (17.69, 41.64)	0.005
Neck angle (°)	33.12 (25.31, 41.80)	32.80 (24.68, 43.27)	0.768
**Vessel diameters [median (IQR)]**			
Coeliac artery (mm)	6.85 (5.96, 7.81)	7.27 (6.44, 8.47)	<0.001
Superior mesenteric artery (mm)	6.50 (5.72, 7.31)	7.44 (6.56, 8.22)	<0.001
Left renal artery (mm)	4.73 (4.05, 5.53)	5.25 (4.57, 5.95)	<0.001
Right renal artery (mm)	4.46 (3.86, 5.15)	4.99 (4.27, 5.63)	<0.001
Left CIA (mm)	12.86 (11.55, 14.15)	17.36 (15.33, 19.91)	<0.001
Right CIA (mm)	13.40 (12.01, 14.84)	17.64 (15.56, 20.32)	<0.001
Left EIA (mm)	8.27 (7.42, 9.14)	10.14 (9.29, 10.98)	<0.001
Right EIA (mm)	8.20 (7.43, 9.10)	10.07 (9.23, 10.98)	<0.001
Left IIA (mm)	6.84 (5.79, 7.91)	9.15 (7.71, 10.53)	<0.001
Right IIA (mm)	6.93 (5.94, 7.99)	8.99 (7.80, 10.48)	<0.001
**CIA stenosis % [median (IQR)]**			
Right CIA	13.93 (10.21, 18.47)	12.49 (8.76, 17.31)	<0.001
Left CIA	11.74 (8.30, 16.94)	10.39 (7.64, 14.49)	<0.001
**Tortuosity index [median (IQR)]**			
Thoracic aorta	1.20 (1.03, 1.29)	1.23 (1.05, 1.31)	<0.001
Neck	1.01 (1.00, 1.02)	1.01 (1.00, 1.02)	0.035
Coeliac artery	1.05 (1.01, 1.10)	1.04 (1.02, 1.10)	0.664
Superior mesenteric artery	1.04 (1.02, 1.08)	1.04 (1.02, 1.07)	0.362
Left renal artery	1.08 (1.04, 1.17)	1.09 (1.04, 1.18)	0.614
Right renal artery	1.10 (1.05, 1.16)	1.11 (1.05, 1.18)	0.256
Infra-renal aorta	1.08 (1.06, 1.12)	1.08 (1.05, 1.12)	0.944
Left CIA	1.08 (1.03, 1.15)	1.07 (1.03, 1.13)	0.026
Right CIA	1.06 (1.03, 1.12)	1.08 (1.03, 1.14)	0.021
Left EIA	1.13 (1.09, 1.20)	1.17 (1.12, 1.25)	<0.001
Right EIA	1.16 (1.10, 1.24)	1.23 (1.15, 1.33)	<0.001
Left IIA	1.12 (1.06, 1.22)	1.12 (1.06, 1.23)	0.544
Right IIA	1.09 (1.04, 1.17)	1.11 (1.05, 1.19)	0.006
**Calcification, cm^3^ [median (IQR)]**			
Neck	0.04 (0.00, 0.17)	0.05 (0.00, 0.20)	0.049
Left renal artery	0.00 (0.00, 0.00)	0.00 (0.00, 0.00)	0.929
Right renal artery	0.00 (0.00, 0.00)	0.00 (0.00, 0.00)	0.555
Left CIA	0.22 (0.06, 0.44)	0.26 (0.09, 0.47)	0.069
Right CIA	0.25 (0.09, 0.50)	0.30 (0.11, 0.57)	0.014
Left IIA	0.04 (0.00, 0.13)	0.06 (0.01, 0.17)	0.001
Right IIA	0.02 (0.00, 0.10)	0.04 (0.00, 0.13)	0.001

CIA, common iliac artery; EIA, external iliac artery; IIA, internal iliac artery; AAA, abdominal aortic aneurysm; IQR, interquartile range.

**Table 4:** Comparison of aortic wall thrombus index and thrombus volumes for Cluster 1 and Cluster 2

	Matched cohorts (ASI)		
	Cluster 1	Cluster 2	*P*-value
*n*	878	577	
**Wall thrombus index [median (IQR)]**			
Thoracic aorta	4.33 (2.77, 7.13)	4.14 (2.60, 6.83)	0.064
Visceral aorta	31.29 (22.01, 41.06)	24.82 (16.00, 34.51)	<0.001
Neck	16.57 (12.00, 22.62)	14.08 (10.94, 19.05)	<0.001
Coeliac artery	4.97 (2.16, 8.86)	4.57 (1.92, 7.52)	0.022
Superior mesenteric artery	4.07 (1.72, 7.73)	4.41 (1.85, 7.64)	0.864
Left renal artery	6.43 (2.30, 12.32)	6.87 (2.34, 13.58)	0.599
Right renal artery	4.86 (1.70, 10.18)	4.55 (1.25, 9.44)	0.162
Infra-renal aorta	51.36 (38.09, 62.75)	42.82 (27.28, 56.67)	<0.001
Left CIA	22.85 (16.70, 31.41)	20.53 (14.87, 28.43)	<0.001
Right CIA	25.08 (18.93, 33.32)	24.27 (16.49, 32.62)	0.027
Left EIA	7.10 (4.16, 12.27)	7.93 (4.41, 12.72)	0.184
Right EIA	7.67 (4.24, 13.19)	7.77 (4.45, 12.82)	0.969
Left IIA	14.97 (6.76, 24.33)	15.20 (8.41, 22.41)	0.455
Right IIA	11.57 (4.80, 20.71)	13.43 (7.17, 22.14)	0.002
**Thrombus volume cm^3^ [median (IQR)]**			
Thoracic aorta	4.50 (2.89, 6.61)	5.26 (3.44, 7.93)	<0.001
Neck	1.83 (1.04, 3.13)	1.96 (1.07, 3.20)	0.366
Infra-renal aorta	96.99 (63.81, 143.66)	94.90 (49.43, 153.24)	0.347
Left CIA	1.32 (0.92, 2.05)	2.28 (1.50, 3.74)	<0.001
Right CIA	1.69 (1.17, 2.46)	2.85 (1.90, 4.23)	<0.001

CIA, common iliac artery; EIA, external iliac artery; IIA, internal iliac artery, aneurysm; IQR, interquartile range.

### Assessment of inter-cluster differences in thromboembolic outcomes

Cluster 1 were observed to have a greater rate of post-operative thromboembolic events (e.g. limb ischaemia, bowel ischaemia, and stroke) 2.6 vs. 1.7%, *P* = 0.35 but event rates were low, and the observed differences were not significant. On univariate analyses, in addition to patient cluster, following variables were found to have an association with post-operative thromboembolic events: ASI, comorbidities [e.g. COPD, chronic kidney disease (CKD)], smoking status, medications (e.g. antiplatelet), symptomatic AAA, treatment centre, treatment modality (open or EVAR), tortuosity of the thoracic aorta and neck, calcification of the neck, superior mesenteric artery (SMA), CIA and IIA, AWT index for the neck, infra-renal aorta, SMA and CIA, and CIA stenosis. Two additional variables [abnormal electrocardiogram (ECG) and statin prescription] were also included due to pre-specified clinical relevance. Subsequent analysis of the effect of cluster on postoperative thromboembolic events, adjusted for co-morbidity, vascular morphology, and thrombus burden, suggested no statistically significant difference in thromboembolic event rates between clusters (OR 1.56 95% CIs 0.71–3.43, *P* = 0.23).

## Discussion

Unsupervised machine learning applied to full automated volume segmentation of AAA imaging generated two distinct morphological subtypes, which mainly differed by their thrombus burden in aneurysmal neck, body, access and visceral vessels. These clusters were sex-imbalanced, with high-thrombus wall index cluster having higher proportion of women. The clusters did not differ in thromboembolic event rate, although these events were few. The emergence of such clusters purely from anatomical features underscores the presence of intrinsic structural heterogeneity within AAA disease that is not captured by conventional diameter-based assessments.

These results highlight that AAA is not a monolithic disease but rather a morphologically and biologically heterogeneous condition. The identified clusters signify that subsets of AAA patients might have different aneurysm structures—for instance, a thrombus-rich, smaller-diameter phenotype vs. a large-calibre, and thrombus-poor phenotype. This concept is reinforced by emerging genetic evidence. Recent large-scale genomic have identified over 100 genetic risk loci for AAA, implicating diverse pathways in its pathogenesis.^26^ The associated genes point to a complex interplay of processes, including lipid metabolism, extracellular matrix remodelling, vascular development, inflammation, and transforming growth factor-β (TGF-β) signalling, each of which might contribute differently to aneurysm formation in different individuals.^26^ Such findings underscore that there may be distinct morphological phenotypes of AAA driven by different predominant mechanisms. Further studies incorporating both molecular and imaging techniques are required to examine these potential endotypes, for example, a subtype with high-thrombus index that might be driven by a pro-thrombotic or inflammatory environment leading to rapid thrombus deposition vs. another endotype that might be driven more by connective tissue degradation and vessel expansion.

Sex differences likely intersect with this heterogeneity. Beyond anatomic measurements, female AAAs have been previously suggested to behave differently at the tissue level: women’s aneurysms tend to grow faster and rupture at smaller diameters than men’s, suggesting potential intrinsic differences in wall biomechanics or remodelling capacity.^27^ Some studies have noted that, even at equal diameter, women’s aneurysm walls may have lower tensile strength or different collagen composition, which could contribute to earlier rupture.^28^ Additionally, hormonal and immunologic factors (such as the loss of oestrogen’s protective effect on vascular inflammation in post-menopausal women) likely play a role in the sex-based divergence in AAA biology.^29^ Women are also more likely to suffer thromboembolic events following AAA repair (e.g. bowel ischaemia and lower limb ischaemia).^30^ Taken together, these observations support a view of AAA as a spectrum of disease influenced by genetic background, sex, and other patient-specific factors. Furthermore, aneurysm morphology may evolve with age, and future longitudinal work is required to understand whether these phenotypes shift over time or represent stable endotypes. Importantly, because our clustering was based exclusively on anatomical variables, age and sex were not inputs to the model, and their observed distribution across clusters should be interpreted as an emergent property rather than a driver of the clustering process.

This study demonstrates the utility of FAVS combined with unsupervised learning to phenotype AAAs. Advanced imaging software now allows rapid, reproducible quantification of aneurysm morphology beyond the aneurysm diameter.^6^ In our dataset, FAVS provided volumetric measures of thrombus, calcification, tortuosity, and conventional metrics of diameters, neck angulation, and other features that would be tedious or prone to observer error if measured manually. By leveraging these rich data with a clustering algorithm, we uncovered potentially novel anatomical subtypes that traditional measurements might overlook. What is more, although the clustering was data-driven, clinical review by vascular surgeons confirmed that the anatomical patterns reflected recognizable phenotypes that could carry practical implications for procedural planning and EVAR complexity. This data-driven approach could in the future augment clinical assessment and move us towards a more personalized or precision-medicine strategy in AAA management. Although the silhouette score of 0.4 suggested moderate cluster separation, the decision to adopt a two-cluster solution was also guided by clinical interpretability. The clustering separated patients primarily along two anatomical axes, iliac access vessel morphology, and neck morphology, which align with known technical considerations in EVAR planning. Further subdivision beyond two clusters did not yield clinically distinct or more stable phenotypes. Current clustering requires further validation, before clinical translation, but even in its current form, provides an insight into how such technology can be leveraged for patient benefit. For instance, identifying that a patient’s aneurysm falls into a ‘high-thrombus, small-vessel’ subtype (akin to Cluster 1) could influence pre-operative planning. Endovascular repair in such a patient may warrant the use of low-profile devices or adjunctive access techniques (e.g. iliac conduits or angioplasty) given the narrow iliac arteries.^31^ The increased thrombus burden might also prompt careful intraoperative imaging and handling to mitigate distal embolization, or even consideration of alternative strategies if thrombus appears mobile. On the other hand, Cluster 2 aneurysm with relatively less thrombus might prioritize concerns about maximal wall stress and rupture risk over embolic risk. In this way, anatomical clustering could in the future inform a tailored approach—selecting the right tools, anticipating complications, and counselling patients based on the particular subtype of AAA they harbour. Such stratification could enable going beyond the one-size-fits-all criterion of diameter >5.5 cm for repair, aligning with contemporary calls for towards individualized vascular care.^32^  *Post hoc* modelling confirmed that sex and age are associated with cluster membership, supporting the notion that biological differences, particularly sex-linked vascular characteristics, influence aneurysm morphology. However, the modest explanatory power (2.6%) of these variables suggests that anatomical heterogeneity extends beyond demographic factors alone. These findings underscore the need for future work integrating demographic, molecular, and anatomical data to refine phenotype definitions and better understand their underlying mechanisms. Interestingly, thrombus metrics emerged as key driver differences between clusters, which suggests that intraluminal thrombus is an important morphological differentiator among AAAs. As automated segmentation and machine-learning tools become more integrated into vascular imaging workflows, they could continually refine such classifications, eventually enabling real-time identification of an aneurysm’s subtype during routine CT evaluation. While thromboembolic and microembolic complications remain clinically important, this study was not powered to evaluate outcome differences, and no statistically significant differences were observed between phenotypes. These findings should therefore be interpreted as exploratory and hypothesis-generating. Larger, prospectively designed studies will be required to investigate whether morphological endotypes influence embolic risk or other clinical outcomes.^33^

Although exploratory, the clustering framework presented here may offer a foundation for future stratification tools in AAA management. Translation into clinical care would require external validation, integration with risk prediction models, and evaluation of procedural and long-term outcomes by cluster type. The morphological phenotypes identified in this study could be incorporated into pre-operative decision-making, through variety of technical considerations such as early use of low-profile endografts, planned iliac conduits, alternative access routes to avoid intraoperative vessel injury, or staged procedures. Integration of cluster classification into CTA reporting or pre-operative planning could provide a rapid anatomical ‘risk map’ to support multidisciplinary discussions, aligning device selection and peri-operative strategy with the specific anatomical challenges posed by each patient. What is more, such a risk stratification could prompt referral to specialist tertiary or quaternary centres for ‘very high risk’ cases, as well as altered peri-operative anticoagulation strategies, or statin dose adjustment. Furthermore, for validation in a larger cohort, successful and supervised ML approaches can be considered to produce a numerical risk score for thromboembolic events, which could be factored into discussions with patients and management strategies. We plan on developing a continuous morphology score and clinically meaningful thresholds and prospectively test it for associations with predefined outcomes and incremental predictive value beyond current criteria. If validated, these phenotypes could inform procedural planning and patient selection in future multi-centre studies and phenotype-guided clinical trials. While this remains an early-stage application, it illustrates a pathway by which unsupervised phenotyping could evolve from an exploratory research tool to a practical adjunct in individualized AAA management.

This study has several limitations. First, our analysis was retrospective and observational, thus it is possible that unmeasured confounding factors influenced both aneurysm morphology and outcomes. It was also limited by the datapoints included in NVR, relative lack of granularity in the type of EVAR device used, technical steps and adjuncts used during procedures, and complication data. Second, the clustering methodology itself has constraints. We selected a two-cluster solution for its clinical interpretability and consistency with known binary contrasts (e.g. high vs. low thrombus burden), but the optimal number of clusters in AAA morphology is not definitively known. The moderate silhouette score of our clustering (∼0.4) indicates only modest separation between the two groups, implying that AAA anatomical features may exist on a continuum rather than in clearly discrete categories. This is further supported by the moderate adjusted Rand index, suggesting cluster stability, but not perfect alignment (0.498). Some aneurysms in our cohort had intermediate features that could plausibly fit in either cluster, reflecting the inherent variability of the disease, that could represent a spectrum, rather than two (or more) distinct risk groups. However, a three-cluster classification shows less discrimination in exploratory analyses, and the clusters demonstrated distinct and clinically plausible profiles, particularly in iliac and neck morphologies. Similar results using hierarchical clustering support the internal consistency of the two-cluster solution within this cohort, although external validation is required for confirming generizability and cluster stability. Future studies might also include additional features (such as biomechanical stress metrics or genetic markers) to see if more distinct subtypes can be identified. An important additional limitation of our approach is that clustering was based entirely on anatomical variables, without incorporating sex, age, or comorbidities. While this allowed us to define purely anatomical phenotypes, it also introduces the potential for residual confounding. As such, the relationship between cluster membership and clinical outcomes should be interpreted as exploratory, although we have attempted to mitigate this through regression. Future studies with larger cohorts should consider multivariable adjustment to delineate the independent contribution of anatomical phenotype. Fourth, although the overall cohort was large, the number of observed thromboembolic events was relatively low (*n* = 31), limiting statistical power to detect meaningful differences between clusters. A *post hoc* power calculation based on the observed event rates (2.6% in Cluster 1 vs. 1.7% in Cluster 2) and sample sizes (*n*_1_ = 878, *n*_2_ = 577) indicated a statistical power of 21.4% at a two-sided alpha of 0.05. This low power underscores that these findings should be viewed as exploratory and hypothesis-generating rather than confirmatory. What is more, the composite definition of thromboembolic events used in this study encompassed outcomes with diverse mechanisms, some of which may not be directly related to aneurysm morphology (e.g. clamp-related limb ischaemia or procedure-associated cerebral events). While this approach aimed to capture the full spectrum of clinically relevant ischaemic complications, it introduces heterogeneity and potential confounding, and any associations observed should therefore be interpreted cautiously. This has also limited the ability to analyse the data using supervised machine-learning methods, without risking model overfitting. Future studies with greater event rates or larger cohorts will be necessary to validate this potential association. Due to exploratory nature of the study and to allow for broader hypothesis generation, we have not applied statistical multiple testing adjustments. As a result, the reported findings may have an increased false discovery rate. It is worth acknowledging that a proportion of scans could not be segmented accurately, despite a correct scanning protocol (*n* = 39/1655, 2.3%). Majority of exclusions (*n* = 161) was due to imaging quality, especially in light of inclusion of cases which have underwent open surgical repair and have not had thin-slice CTA required for segmentation, yet segmentation failure reflects current limitations of FAVS algorithms. This challenge is increasingly recognized in the field of FAVS, as shown in the recent study by Van Tongeren *et al*.,^34^ who demonstrated the need for continued refinement of both imaging protocols and software performance. Finally, our analysis focused on anatomical and volumetric data from CT imaging; we did not incorporate further radiomic features into the analysis, which could have proved informative when looking into thrombus morphology.

## Conclusions

Unsupervised machine learning applied to fully automated imaging data can identify distinct abdominal aortic aneurysm morphological phenotypes characterized by differences in thrombus burden, vessel calibre, and sex distribution. These phenotypes emerge independently of clinical variables and highlight the underlying anatomical heterogeneity of AAA disease. Although no statistically significant differences in thromboembolic outcomes were observed, this approach provides a framework for future studies aimed at integrating anatomical phenotyping with clinical, molecular, and biomechanical data. Further work is needed to determine whether such phenotypes have predictive or therapeutic relevance in AAA management.

## Supplementary Material

ztaf136_Supplementary_Data

## Data Availability

The summary data underlying this article will be shared on reasonable request to the corresponding author.

## Lead author biography

Mr Michal Kawka is an NIHR Academic Clinical Fellow in Vascular Surgery at City St George’s University of London. He was graduated from Imperial College London in 2023. His research interests lie in the use of data, artificial intelligence for clinical outcomes research in aortic, peripheral arterial diseases, and evaluation of novel surgical technologies.
